# History of the Medical Department of the University of Louisville: An Introductory Lecture, Delivered Nov. 1st, 1852

**Published:** 1853-01

**Authors:** Lunsford P. Yandell

**Affiliations:** Professor of Physiology and Pathological Anatomy


### WESTERN JOURNAL

OF

### MEDICINE AND SURGERY.

JANUARY, 1853.

Art. I.—*History of the Medical Department of the University of Louisville: An Introductory Lecture, delivered Nov. 1st,* 1852. By Lunsford P. Yandell, M. D., Professor of Physiology and Pathological Anatomy.

Gentlemen:

In behalf of my colleagues, for whom I appear before you, this evening, I bid you welcome to our City and this Institution. We greet you cordially as friends, and tender to you our hospitality and friendly offices. We pledge you our unwearied efforts to render your sojourn in Louisville profitable and agreeable. You have come, many of you, from far, leaving behind you happy homes and loving friends. The absence of these we cannot hope entirely to supply; but the remembrance of what you have given up for the sake of your profession will inspire us with deeper solicitude for your welfare; and we shall labor the more zealously so to inflame your minds with the love of science that this passion may replace, for a season, all repinings towards home. You have a laborious winter before you; you have made choice of a laborious profession. It will be our aim and study to render the toilsome path upon which you have entered a pleasant one; to strew along it, if we may, an occasional flower, or at least bring the light of our enlarged science fully to illuminate it, and conduct you along it so prosperously and safely, that, in looking back hereafter upon the happy and useful hours of your lives, you shall recall this season as one well spent.

I am to speak to you on this occasion of the Institution in which you are at present assembled. I propose to give you some account of the origin, progress, and present condition of the Medical Department of the University of Louisville. I do not purpose to write its history in detail, nor to pronounce upon it any studied eulogy; but to recite briefly some of the most prominent events in its career; to declare the policy by which it has been guided; and to refer to some of the reasons which, in the opinion of its Trustees and Medical Faculty, entitle it to look to the profession for its sanction and continued support. Such a narrative, I flatter myself, will not be uninteresting to you its pupils; while such a report, now that the school is entering upon its sixteenth session, seems to be due to the citizens of Louisville, whose liberality in endowing it, gives them a claim to know what it has achieved or attempted, and what are its present condition and future prospects.

Of the three professions commonly styled "learned," Medicine alone rests upon observation and experience. Law and Theology are historical and dogmatic. The profound jurist is made by the books of his profession. His business is with precedents and the authorities in law. With him reading is everything, and, other things being equal, his eminence will be in proportion to the extent to which he has stored his mind with legal precepts and decisions. Experience and observation are of great avail in the practice of the law, but they have no part in the education of the lawyer.—So in Theology:—all is founded upon authority. In every question involved in this sublime science, the appeal lies to the Bible. With the Bible alone, the student, possessed of a good mind, and shut up to himself and without aid from any one, might frame a perfect system of theology. His observation, however varied and accurate, his experience, however deep, would not enable him to enrich or adorn it with any new fact or principle. It must all come from that only source of heavenly light—the volume of revelation.

But with medicine it is otherwise. In our profession, authority is worth but little. We have no traditions or decisions that have the binding force of law—no authorities from which we cannot appeal—no records of infallible wisdom, but the book of nature. Medicine is experimental and demonstrative; it consists of phenomena, which must be seen, scrutinized, and pondered upon. The eye of the naturalist, the laboratory of the chemist, and the knife of the anatomist are all requisite to its advancement. Books are of the last importance to the student of medicine, but books alone could never make a thorough physician; they could never impart a knowledge of the taste of opium, or the color of chlorine;—they could not teach a student how to determine whether a blister had drawn well, or how a patient looked with measles or smallpox. He is obliged to see and observe as well as read. He appeals not to the fathers in medicine for any fact in human anatomy, but repairs to a more infallible source of knowledge; he is content with nothing short of a demonstration.

This, if I am not mistaken, is the principle upon which medical schools have obtained such a currency, and are so much relied upon in the acquirement of a medical education. Students of law and divinity no doubt derive important advantages from the lectures upon which they attend on law and theology, but to the medical student schools of medicine are indispensable. They have had an existence in every age of the healing art. Six hundred years before the Christian era we are told that three were founded by the Asclepiades, and two centuries later Hippocrates gave public instructions in anatomy and the treatment of disease, and labored to disseminate a knowledge of medicine. The great school of Alexandria was founded three hundred years before the birth of Christ; and it was there that Galen, in the fifth century of its existence, inspected one of the two human skeletons that formed the basis of his book on Anatomy. The progress of these primitive schools was slow; nor was there in those which succeeded them in Europe, at a later day, any principle of vigorous growth. When so many other departments of human knowledge were rapidly advancing, the medical teacher was still contented servilely to copy and communicate what had come down to him from the early Greek and Arabian physicians.

Our country was slow to embark in the medical instruction of her own sons. A century and a half after the colonies were settled the medical students of America were still obliged to repair to the colleges of Europe for the completion of their studies. It was not until the University of Edinburgh had been attracting scores of young American physicians across the sea for forty years, that any serious effort was made to establish a medical school on our continent. This honor belongs to Dr. John Morgan, who, by an address remarkable for its earnest and sound argument, prevailed upon the trustees of the college of Philadelphia to found the institution now represented by the University of Pennsylvania. This first American school of medicine was organized in 1765, while Dr. Franklin presided over the College. Three years afterwards a similar institution was founded in New York, but failed to command the success which has attended the Philadelphia school. A medical faculty was appointed in 1782 to give lectures on the different branches of medicine in Harvard University; and in 1804 Dr. John B. Davidge laid the foundation of the medical school at Baltimore. He had returned a few years previously from the University of Edinburgh, where he formed the resolution, in common with his fellow-students, Dr. Hosack, and Dr. Samuel Brown, of establishing a medical school in his native country. I have heard him relate, that the project appeared to the students of the old country extremely absurd, and they made great sport of the embryo professors of America. The opening of his enterprise was anything but auspicious; his first class numbered only six, and his second had but one addition to it. The rise of the other early American schools, though not quite so gradual as that of my old preceptor and friend, was by no means rapid when compared with those of our day. It remained for the West fully to develope the activity of such institutions.

The tide of immigration had been pouring into the Valley of the Mississippi for more than thirty years, and the Western States were still without a medical school. Such students as could afford the necessary means resorted to the Atlantic colleges; those who were unable to incur the expense entered upon the practice of their profession without the advantages of public instruction. Kentucky, the pioneer of the new states, took the lead in medical education. With whom the thought of founding a medical college in Lexington first originated, it is perhaps impossible now to ascertain, but as early as 1816 some steps had been taken in that direction. In that year lectures were delivered by Dr. Wm. H. Richardson while yet an under-graduate in medicine. In 1817 he was associated with Dr. Benjamin W. Dudley, Dr. Daniel Drake, Dr. James Blythe, and Dr. James Overton, in the Faculty of the Medical Department of Transylvania University. These gentlemen delivered a course of lectures to a class of twenty students, of whom Dr. W. L. Sutton, the first President of the Kentucky Medical Society, is one of the surviving members. The result of this enterprise does not appear to have been satisfactory; troubles originated in the Faculty, and the school was suspended after a single session.

In the summer of 1819 the Faculty was re-organized, Dr. Charles Caldwell and Dr. Samuel Brown taking the place of Dr. Drake and Dr. Overton, the first of whom had in the meantime returned to Cincinnati, and the latter had removed to Nashville. Dr. Caldwell brought with him from Philadelphia a high reputation both as a writer and a lecturer. Dr. Brown was a man of showy parts, of varied learning, of fine person, and elegant address. Dr. Dudley had already given promise of that rare surgical skill which has since rendered him so distinguished. Dr. Richardson had the reputation of being a successful practitioner of obstetrics, and was recommended by cordial and popular manners. Dr. Blythe, the professor of Chemistry, was a learned Presbyterian clergyman, and his connection with the school was calculated to conciliate that large and influential body of christians.

Under the direction of a Faculty thus constituted it became at once manifest, that the Medical Department of Transylvania University was soon to exhibit an example of prosperity at that time unparalleled in the history of medical schools. Its location had great advantages for the time. Lexington, from its literary eminence, had acquired the title of "*Athens of the West.*" It was the commercial as well as the literary emporium of the Western States. The late Dr. Horace Holley, at that time President of the University, with powers of display seldom equalled, conferred upon the institution a remarkable lustre. The medical school was a new enterprise and had in it all the excitement of novelty and hope. Its Faculty was ardent, zealous, and gifted. It was situated in the midst of a wide country rapidly increasing in population. The first session of the school opened with a class of 37 pupils; its second class numbered 93; its third 138; its fourth, 171. Before the commencement of the fifth session, Dr. Drake, who had made an abortive effort to found a similar institution in Cincinnati, was united to the Faculty, as Professor of Materia Medica. The number of the succeeding class was 200; and that of the sixth, 234. At the end of this session, Dr. Brown resigned the chair of Theory and

Practice of Medicine, to which Dr. Drake was transferred, and Dr. Charles W. Short was elected Professor of Materia Medica and Medical Botany. The ensuing class, in the autumn of 1825, numbered 282 students. The one which followed was not so large, and the next declined to 190. At the termination of this session, Dr. Drake resigned his professorship. In the summer of 1827, Dr. John E. Cooke, who had attracted the attention of the profession by some able papers in the *Medical Recorder*, and by his "Pathology and Therapeutics," the first volume of which had just been published, was invited from Winchester, Virginia, to the chair of Theory and Practice. The number of students the following winter was only 150; but the next session exhibited an increase, and for several years the classes continued steadily to grow. In the spring of 1831, Dr. Blythe resigned the chair of Chemistry, and the writer of this narrative was appointed his successor, with the late Mr. H. Hulbert Eaton as Assistant. Unfortunately for science, this promising young man was cut off after participating in a single course of lectures, dying of pulmonary consumption in the 23d year of his age.

The institution in 1835 was again in a highly flourishing condition. Its classes had risen above 260. To the eye of the common observer all about it gave promise of stability; but appearances were deceptive, and in the midst of such success the conviction was forced upon the minds of the Faculty, that the school had filled up the measure of its usefulness. Lexington, the most eligible site for a medical school when this was organized, was now admitted to be deficient in some of the elements essential to the establishment of a great and enduring institution. With the advancement of medical science in our country it had ceased to be able to satisfy the demands of the profession. It had no hospital, and furnished very precarious and inadequate means for anatomical study. In the winter of 1835–6 it came to be felt and acknowledged by the Faculty that the department, if it was to be maintained in the position of ascendency which it had enjoyed from the beginning, must be transferred to a situation possessing more advantages than were then afforded by that beautiful city. Louisville suggested itself to the mind of every member as a point combining all the facilities for a medical school, and accordingly in the spring of 1836 it was resolved, with entire unanimity, to attempt to remove it to this place.

When the period for carrying out this resolution arrived, it was ascertained that the measure was impracticable; the professors might remove to Louisville, but the citizens of Lexington and the Trustees of the University would not entertain the proposition to transfer the Medical department. The agitation of the question led to dissentions among the professors, and finally to a dissolution of the Faculty; three of the members, Dr. Dudley, Dr. Richardson, and Dr. Short remaining in Lexington, Dr. Caldwell, Dr. Cooke and myself accepting places in the *Louisville Medical Institute.*

The Medical Institute of Louisville was chartered by the Legislature of Kentucky on the 2d of February, 1833, and various attempts were made without success to put it in operation; at length the citizens becoming interested in the project, a town meeting was held, at which on the 30th of March, 1837, it was resolved that there ought to be a college in the city of Louisville, with *Medical* and *Law Departments*, and that it was expedient that the Mayor and Council should proceed at once to endow the first of these.

The Mayor and Council were prompted by these resolutions of the citizens to grant the square bounded by Eighth and Ninth, and Chestnut and Magazine Streets, t[illegible] the Managers of the Medical Institute; and they furthe[illegible] resolved to erect necessary buildings for a Medical schoo[illegible]

at a cost not to exceed $30,000, and to advance in cash for the purchase of a library, anatomical museum, and the requisite apparatus, an additional sum of $20,000. On the 11th of April the Board met, and accepted the donation of the city. During the summer six professorships were filled, Dr. Miller, who had resigned his chair in order that the Board might be entirly unembarrassed in making their new arrangements, being appointed to the chair of Obstetrics, Dr. Cobb to that of Anatomy, and Dr. Joshua B. Flint to that of Surgery. To Dr. Caldwell, Dr. Cooke and myself were assigned the chairs which we had respectively held in Transylvania University, namely, Institutes of Medicine, Theory and Practice, and Chemistry. Subsequently I was transferred to the chair of Materia Medica and for one season delivered lectures on that branch as well as Chemistry.

The first course of lectures in the Medical Institute was delivered in the upper rooms of the City Work-house, which stood upon the site of our present edifice, to a class of 80 students. The appearance and appointments of the old structure in which we were to commence our labors were unattractive, straitened, and comfortless enough; and now as I look back upon the new enterprise I can see that there were discouragements attending it which might justify the misgivings of many of its friends. The Lexingington school was again fully organized. The citizens were roused by the attempt to transfer to a rival city an institution which had been so long a cherished object of their pride, and were resolved to sustain it. Dr. Eberle, at that day one the most popular authors and teachers in the country, had been induced to leave Cincinnati and accept the chair of Theory and Practice of Medicine. One half of the Faculty which had reared the school and conferred upon it a full share of its reputation, still remained identified with it. It had a widely extended, influential, and devoted corps of *alumni* upon which it could rely, and it had a name among the medical institutions of the country which the success of nineteen winters had been constantly strengthening and extending.

Such was the School in the face of which the Medical Institute of Louisville was to rise; nor was Transylvania the only powerful rival in its neighborhood. The Ohio Medical College, though crippled by the withdrawal of Dr. Eberle and Dr. Cobb, was again organized, and with many other advantages could boast of a reputation as ancient as that of the sister institution at Lexington. The Cincinnati Medical College was also contending vigorously for the first rank among Western Medical Schools, and when I tell you that Dr. Drake, Dr. Parker, of New York, Dr. McDowell of St. Louis, the late Dr. Harrison, of Cincinnati, and Dr. James B. Rogers, of Philadelphia, Dr. Rives, now of the Ohio Medical College, and my colleague, Dr. Gross, composed its Faculty, you can judge with what chances of success. Not a few of our friends were desponding. It was doubted whether we should have any students at the time we proposed to commence our first course, and some of us were kindly advised to give up the project as hopeless.

But not so thought the Faculty. To their minds it was evident that the enterprise must prosper. It could not be doubted that Louisville, from its geographical position and many other natural advantages, must become the seat of a great medical school, and the citizens had wisely decreed the means necessary to its establishment.

Our first class, I have mentioned, numbered 80 students, of whom 27 received the degree of M. D. in the spring. The class at Lexington numbered 230, which was only about twelve short of the preceding class.

It was a noble effort to found the first medical school in the West. It placed a liberal medical education within the reach of hundreds of meritorious young men who must otherwise have grown old in their profession without its advantages. The Transylvania medical school was a source of substantial blessings to the country. They who founded it and by their labors gave to it its brilliant reputation, were pioneers in medical education, benefactors of their profession and their race, and as such their names will live in the memories of men.

Those who came to establish the medical school at Louisville were also pioneers. They were still bearing forward the light of our beneficent science in the direction in which the "star of empire" has so long held its way. When the steeple which surmounts this edifice was erected, it was the last reared in honor of medicine upon which the sun shone in his journey down the evening sky—the first to greet the traveler coming from the "far west." Now it is one of the old schools; so rapidly do such institutions grow up in our progressive country.

On the 22d of February, 1838, the corner stone of this building was laid with Masonic honors, in presence of a great concourse of citizens, and the second course of lectures was delivered in these rooms. At the close of the first session, it appearing desirable to fill the vacancy in the Faculty by the introduction of Dr. Short, who had again after the dissolution of the Faculty accepted a chair in the Lexington school, I resigned the professorship of Materia Medica and was appointed by the Board of Trustees to the chair of Chemistry. The election of Dr. Short completed the organization of the Institute. A member of the Faculty was commissioned by the Trustees to visit Europe for the purpose of increasing the library, chemical apparatus, anatomical models and preparations, and other materials of illustration for the school. The second session opened under favorable circumstances. The new and splendid edifice presented a strong contrast to the old rooms in which the incipient exercises of the institution were performed; and the fine library and suites of apparatus arrived from Europe in good season to render the preparation for teaching the several branches complete. The second class numbered 120.

In the summer of 1839, the Cincinnati Medical College suspended operations, and Dr. Drake, its founder, was elected Professor of Clinical Medicine and Pathological Anatomy, in the Medical Institute, a chair created by the Board of Trustees, on the recommendation of the Faculty, for the purpose of securing the services of that experienced and able teacher. It is worthy of remark that although the effect of this innovation was to raise the tuition fees of the Institute above those of all the neighboring schools, it caused no abatement, but rather an increase, in the ratio of its growth. The number of its third class was 205. At the end of this session Dr. Joshua B. Flint retired from the school, and was succeeded in the chair of Surgery by its present incumbent, Dr. Samuel D. Gross.

The class had now grown to be so large that the usual mode of giving clinical instruction—the students following the professors through the wards of the hospital, and catching, as they could, the remarks made at the bedsides of the patients—was found to be ineffectual; and in order that this most important branch of medical teaching might be rendered efficient and useful, the Faculty determined, with the consent of the city council, to erect a clinical theatre adjoining the Marine Hospital. The following course of lectures was delivered to 209 students, and no portion of it was more satisfactory than that which was given in the clinical amphitheatre. The effect of the improvement was felt to be most salutary. The succeeding class numbered 268.

In consequence of the embarrassed state of the country, the number of students declined the ensuing session, and was only 190; but the institution soon recovered from the temporary depression and the following years exhibited a rapid increase. Its sixth class reached 246; its seventh, 290; and its eighth, 347. It was now confessedly ahead of all the neighboring schools, and probably behind none in the country except the two principal schools of Philadelphia.

During the winter of 1843–4, Dr. Cooke, who had retired to a farm in the neighborhood of Louisville, gave notice to the Board of Managers that he should vacate his chair in the spring, a step which his declining health shortly afterwards would have rendered necessary. He was the first of those who had taken part in the organization of the school to resign his seat in it. The peculiar medical theories and practice of this original man have been extensively commented upon, and are known to every one who has read much of American medicine. Whatever may be the judgment of medical men concerning these, there can be among those who have known him intimately but one opinion as to the purity and excellence of his character. However mistaken he may have been in any of his views, no one ever doubted his sincerity. No one ever associated long with him without the conviction that he was a just, upright, and thoroughly honest man. The feeble state of his health has compelled him entirely to abandon his profession, and for several years past he has lived on his farm, in Trimble county, on the banks of the Ohio.

In February, 1845, during its eighth session, the Legislature of Kentucky granted a charter for the *University of Louisville*, of which the Medical Institute was constituted the Medical department. By the provisions of the charter, the Board of Trustees were to be elected by the City Council, and to hold office for a limited period, instead of filling their own vacancies, and continuing in office for life, as under the original charter. The first class that assembled in the Medical department of the University of Louisville numbered 353 Students, and the second rose to 406. This was in 1847, ten years from the commencement of the enterprise, and I suppose I am safe in saying, that no medical school ever attracted so many students in so short a time. The number, the ensuing session, was 333.

Extensive changes in the Faculty took place after the close of this session. In February, 1849, Dr. Drake signified to the Board of Trustees that he should resign his professorship at the end of the term. Later in the season the chair held by Dr. Caldwell was vacated; and in June, Dr. Short carried into effect a wish which he had long indulged, of retiring from the turmoil which seems to be inseparable from medical schools. These professors were all men experienced, learned, and widely known. Dr. Caldwell was for many years one of the chief ornaments of Transylvania University, and by his energy and industry, his great learning, and his eloquence, had contributed a full share to its rapid rise and wide popularity. He was far more actively concerned than any of his colleagues in procuring from the city of Louisville the noble endowment of the Medical Institute, and his reputation for learning and originality had been of the greatest service to the institution in its earlier years. Dr. Drake was at the height of his popularity, and in the full maturity of his intellect. As a lecturer or writer, he had made himself known to every educated American physician. With an unfailing zeal in his profession, untiring industry, a mind singularly active, vigorous, and comprehensive, and an eloquence which never failed to excite and gratify the interest of his pupils, he would have taken a high rank in any medical school. Dr. Short differed in the character of his mind from both his distinguished colleagues, but possessed qualities which rendered him a most valuable officer. His high scientific attainments, the soundness of his judgment, his dignity and urbanity of manners, his amiable temper, and blameless life, added character and weight to the institution.

These eminent teachers were succeeded by Dr. Elisha Bartlett, Dr. Lewis Rogers, and Dr. Benjamin Silliman, Jr., the latter in the chair of Chemistry, the Board of Trustees having done me the honor to assign to me the department of Physiology and Pathological Anatomy. The influence of so extensive a revolution was feared by some, but the sequel proved that the institution had become sufficiently established in the confidence of the public to bear the change without loss. The number of the succeeding class was 376—a gain of more than forty upon the one of the previous year, and the largest but one ever attracted to the University.

The prospects of the school were never brighter than they appeared to be at the close of that session. There was not a speck to be descried upon its horizon in any direction. Its faculty was united and harmonious; its pupils had retired to their homes in the most favorable temper; it had been now for several years far in advance of all the western schools;—all the omens were auspicious. But before the opening of another collegiate year, the Trustees were called upon to fill two vacancies in the faculty. Dr. Bartlett and Dr. Gross, late in the summer of 1850, resigned their places, and accepted chairs in the University of New York. Dr. Drake was recalled by the Board to the professorship which he had formerly held, and Dr. Gross was succeeded by Dr. Paul F. Eve, of the Georgia Medical College, at Augusta. The number of students the session ensuing was 282.

At the close of his first course of lectures in New York, Dr. Gross returned to Louisville, and Dr. Eve resigned the chair of Surgery in his favor. Dr. Gross was re-elected in 1851, and Dr. Eve, who had generously relinquished a place to which he felt that his friend had stronger claims, was invited to a chair in the medical school about to be organised at Nashville. The number of the class, as you are aware, was 262.

The friends of the Ohio Medical College, being desirous of reorganizing that institution, prevailed upon Dr. Drake, last spring, to return once more to the chair in that school of which he was the first occupant. Dr. Cobb, who was also for many years one of the ornaments of that institution, was induced to sever his connection with the University and accept the professorship in which he made his debut as a medical instructor. The successors of these distinguished and popular teachers, Dr. Austin Flint, and Dr. Benjamin R. Palmer, are before you this evening.

In reviewing this rapid sketch of the origin and progress of the medical schools of Kentucky, one of the first facts that would impress the mind is, that of the five professors who took part in the first course of lectures, although more than the third of a century has since passed away, all but two still survive; and that of the seven who constituted the two first faculties, more than half are now living. Dr. Brown was the first to pay the debt of nature. He died in Alabama, in 1830. Dr. Blythe followed him in 1841, both having retired from the school long anterior to the period of their death. Dr. Richardson died in September, 1845, while still a professor in Transylvania University. Of the ten individuals who were at different times connected with that institution in the twenty years from 1817 to 1837, all but the three mentioned still live. In the fifteen years during which the Louisville school has been in operation, its Faculty has been the subject of very numerous changes; a dozen chairs have been vacated, from first to last; fourteen persons have held professorships in the school, of whom, up to this hour, not one is numbered with the dead.* These vital statistics, I have

---

* Four days after these words were pronounced, Dr. Drake, one of the oldest and one of the most distinguished of all those who have particisupposed would be interesting to you, and may have some interest for the committee on Hygiene appointed by our State Medical Society. They go far to prove, I think, that medical schools, with all their attendant troubles, labor, and perplexities, are not unfavorable to longevity.

This historical sketch would be incomplete without some reference to the adverse circumstances with which this institution has had to contend. It will strike you, no doubt, as paradoxical but it is nevertheless a serious truth, that one of the difficulties which beset it at a very early period, was its great prosperity. When first established, and while struggling for existence, it had the sympathy and good wishes of all the people of Louisville; but as it grew rapidly, and from being a doubtful enterprise, came to be a most flourishing institution, it began to be urged by some, that the professorships were too remunerative. A plan was accordingly set on foot to limit the receipts of the professors. The idea of taxation was a plausible one, and some of the wisest and most judicious citizens fell in with the project. But in order to effect it, it was necessary to obtain a modification of the charter, for the original Board of Managers was a "close corporation," and the members were opposed to the new scheme. In furtherance of this design, an effort was made to transfer the Institute from this Board to the Board of Trustees of the Louisville College, who were elected by the City Council. The attempt did not succeed, but the project was never abandoned by its authors, and at length the charter for a University having been obtained, it was supposed that under it the measure might be accomplished. But this also failed.

---

pated in these schools, died at Cincinnati, in the 68th year of his age. The disease which terminated his useful career was congestion of the brain, brought on, there is reason to believe, by too intense mental application, and the deep solicitude and anxiety for the success of his favorite school, naturally excited by the circumstances under which he had just then become connected with it.

It was ascertained that according to the provisions of this charter the fees of one department could not be taken and applied to the support of professors in another. Finally, a new city charter was framed, and by a clause in this charter the government and property of the University were transferred from the Board of Trustees elected by the city council to a Board to be elected by the people.

The grounds upon which the Trustees resisted taxation I have not time to state at full length, but they were substantially the following: 1st. That the city, in making a *donation* for a University, did not expect any return in the shape of rents on its property. 2dly. That it did not appear to be equitable or just, that the earnings of one set of professors should be appropriated by those in another department. 3dly. That in the progress of medical science and the increasing competition of medical schools, all the receipts of the department would be necessary to keep it in an advanced position, by increasing its library, apparatus, anatomical models and preparations, and the other means of instruction demanded by a medical school. These considerations must be admitted, I think, to possess great weight. As guardians of a public institution in which the city feels a natural pride, they could not consent to any measure which would jeopard its prosperity or impair its usefulness. They felt bound to husband its resources and so to apply its revenues, as to fix it upon a broad and secure foundation. The citizens of Louisville, when they voted the large appropriation, had in view the establishment of a school that should afford to medical students all the advantages claimed by any similar school in the country, and the Board were resolved that, with their consent, these expectations should not be disappointed.

But the agitation to which the proposition to tax the medical professors gave rise, and which has been kept up now for more than twelve years, has been eminently prejudicial to the institution. It has had the effect of weakening the attachment of professors to it, by inspiring them with distrust of its stability. Each successive season, as the scheme has been brought up, in some new phase, apprehensions of change were excited. Revolution was continually threatened. The feeling was communicated to the pupils. They heard, in their boarding houses, and read in the daily papers, that the school was in danger, and often left the institution in the spring with strong doubts as to what was to be its fate. Nor were there wanting elsewhere those interested in seeing it decline, who took advantage of these rumors and exaggerated the perils to which it was exposed.

Fortunately this question is approaching its final settlement. The act of the Legislature creating the city charter provided that the constitutionality of the clause relating to the University should be submitted to our courts of law ; and the case is now under adjudication. One tribunal has decided against the new charter and in favor of the former organization. The cause has been carried up to the Appellate Court of the Commonwealth where it has been argued, and we shall no doubt have a decision before the close of our term. If confirmatory of the former decision, we may hope that this agitation will cease and determine forever.

Before leaving this subject I wish to say, that no censure is meant to be cast upon those who have so strenuously urged taxation. No doubt, they have been fully persuaded of the policy and justice of that measure; they have believed it to be practicable and proper. To the great mass of its advocates, I am persuaded, nothing has appeared more fair, equitable, or judicious. They are really the friends of the school. They wished it complete and eminent success. But I must be permitted to add, that its worst enemy could hardly have devised a scheme fraught with more mischief than the one proposed. If he had gone about its ruin, he could not have more successfully compassed it than by those measures which at one time threatened to prevail. That the growth of the school has not been seriously impeded by this agitation so long kept up, only shows how firmly it is rooted in the confidence of the profession.

The contributions of an institution like this to the literature of the profession, form an interesting and imporant part of its history. The University of Louisville, in this respect, will be found, I believe, to compare not disadvantageously with any medical school in our country. The treatise of Dr. Cooke on Pathology and Therapeutics, in two thick octavo volumes, a work certainly of no ordinary ability, was published before the author entered the Louisville school and can hardly, therefore, be included among the books to which the school has given origin; but the great work on the Principal Diseases of the Interior Valley of North America was composed by Dr. Drake while connected with the University. A volume on Human Parturition has been prepared by the Professor of Obstetrics, which has received the highest encomiums of the medical press at home and abroad. The Professor of Surgery, since he came into the school, has revised a new edition of his elaborate work on Pathological Anatomy, written an original monograph on Wounds of the Intestines, and composed a Treatise on the Urinary Organs, which a leading medical journal of England pronounces the ablest yet written upon the subject. The Professor of Chemistry has prepared a popular text-book, embracing a clear and succinct view of the present state of that science. The Report on Continued Fevers, by the Professor of Theory and Practice, which has hardly yet had time to reach our frontier settlements, is receiving the commendations of all the medical journals, as a work which reflects honor upon the professional literature of our country. A Medical Journal, originated by the school, and conducted by a member of the Faculty, has been in progress thirteen years, and now extends to twenty-six volumes. I might enlarge upon the influence of these works upon the American profession. It would not be difficult to show that they have contributed some part towards elevating its character and extending its fame; but this is a topic upon which I must not venture.

I ought not to pass by entirely without remark the benefits which the University has conferred upon the City of Louisville. I will not go into an estimate of its pecuniary returns;—these are easily calculated. It need only be stated that large classes have been collected by the school for fifteen years, to render it evident that it has brought much money to the city. If the sum be set down at a hundred thousand dollars a year, then the aggregate amount already received from it is a million and a half. But I pass this by as the lowest and least of the advantages which the city has derived from its establishment. It has given Louisville a name among the cities of the world where science is cultivated. It has extended her intercourse with all the parts of our great interior valley, increasing her commerce, and stimulating the industry and enterprise of her citizens. Added to this, is the humanizing influence of science and letters, which it is impossible to estimate—an influence, like that of the sun, quiet, noiseless, but pervading, vivifying, powerful and lasting. How much the school has done to diffuse among all classes a taste for knowledge and science; how much to elevate and improve the tone of society—to direct the minds of our youth towards noble objects and into pure channels of thought and inquiry, can only be known after the coming of other generations. It is at least safe to say, that in these points the University, thus far, has not come short of the expectations of its founders.

A little more than a century ago medical teaching was nearly all addressed to the ear. It was to listen to the prelections of Boerhaave that medical students from all parts of the civilized world flocked to Leyden. It was to hear Cullen, and the Monros, and Black, that they repaired in as great numbers, at a later period, to the University of Edinburgh. The lectures of those days were carefully written out by the professors in their closets and read to their pupils. The teacher of anatomy read in the hearing of his class a description of bones and organs, of muscles and blood vessels; and the chemist read an account of the oxygen, and chlorine, and hydrogen, which he kept closely sealed up in his bottles. These descriptions, unaided by experiment or demonstration, the student must retain in his memory. Long, operose formulas and recipes were to be treasured up, and even ponderous volumes learned by heart.

But all this has given way to the method of demonstration—a method in which the eye is addressed as well as the ear, and the memory while taxed by the increasing fund of scientific knowledge, is yet assisted in its severe task by specimens, models, illustrations, and experiments. The desire and purpose of the instructor is now to demonstrate all that is susceptible of demonstration, and art is put in requisition to represent in form and color whatever pertains to the animal body in health or disease. Hospitals enter as an essential element into this system. There must also be abundant facilities for the study of anatomy. Extensive cabinets, embracing the articles of the materia medica; models, illustrative of healthy, morbid, and comparative anatomy; specimens prepared by the anatomist to exhibit organs in their normal condition and as altered by disease—must be provided. The laboratory must not be dwarfed, but should be on a scale corresponding to the advanced state of chemical science, which daily becomes more and more intimately connected with medicine. And to all the rest must be added the library. There must be books for the teacher, and books for the inquisitive student—not merely the ordinary text-books and systems, but rare monographs, expensive, elaborate treatises, and serials containing the recorded observation and experience of the profession in all countries–works for research to form scholars and authors.

The officers of this institution feel the force of these requirements. They appreciate the responsibility of their position. It has been their endeavor to place the school on a level, in these respects, with the best medical colleges of the day. It is not pretended that all has yet been done that it is desirable to do ; but they would have the past accepted as an earnest of what may be accomplished in future by perseverance in the present policy of the institution. The library will be steadily augmented. New models, and apparatus, and specimens will be added, as art multiplies the resources of the teacher. The *Clinic* connected with the University, will add to the opportunities for the study of disease afforded by the Marine Hospital, and with the growth of our city we may soon expect to have opened to us new and better theatres for clinical observation. When, if ever, the school is suffered to stand still, let its halls be deserted. If, in this age of unparalleled progress, it fails to keep pace with the march of science and to render available to its pupils all the discoveries in our benign art, let it be forsaken of its friends. It asks no patronage or public favor, if, on the most rigid scrutiny, its claims are not found equal to those of any of its sister institutions.

We take but a narrow and unworthy view of such an institution when we regard it only in its relations to the present time and our own generation. We have seen merely the beginning of it. It is still in its infancy. The foundation, we believe, is an enduring one. The commencement of a noble library and splendid museums has been made, but their completion is the work of many years, if indeed libraries and museums can be said ever to be complete. Let us hope that the school, thus richly furnished and endowed, will endure and continue to grow, proving a benefit and an honor to our country in ages yet to come. Hither may repair the medical scholar, to pursue his researches amid the accumulated treasures of the medical minds of all nations and times. The zoologist may come here to study, in our forming cabinet of natural history, the fauna of our region, and the botanist to look into its teeming and beautiful flora. Our collection of organic remains will attract hither the paleontologist of other countries, to inquire into the forms, and structure, and habits of those strange by-gone creations which were once the only tenants of the region where we dwell, and whose bodies are now hardened into stone. Students of medicine from every quarter of the Republic will assemble annually within these walls, to avail themselves of the increasing stores of knowledge afforded by the institution; and some of you after the lapse of half a century, it may be, will listen with satisfaction and pride to your ardent pupils telling on their return home from the University, of the great improvements in the healing art taught and illustrated in the halls of your *alma mater*. Some of these, may we not hope, will be made by you? and thus shall we give to the world the best proof of our ability to teach by sending forth alumni to become discoverers in the profession.

